# Isorhamnetin Alleviates Mitochondrial Injury in Severe Acute Pancreatitis via Modulation of KDM5B/HtrA2 Signaling Pathway

**DOI:** 10.3390/ijms25073784

**Published:** 2024-03-28

**Authors:** Xiaojuan Li, Tao Wang, Qilong Zhou, Fan Li, Ting Liu, Kun Zhang, Ao Wen, Lijuan Feng, Xiaoling Shu, Simin Tian, Yijiang Liu, Yu Gao, Qing Xia, Guang Xin, Wen Huang

**Affiliations:** West China Center of Excellence for Pancreatitis, Institute of Integrated Traditional Chinese and Western Medicine, Natural and Biomimetic Medicine Research Center, Tissue-Orientated Property of Chinese Medicine Key Laboratory of Sichuan Province, West China School of Medicine, West China Hospital, Sichuan University, Chengdu 610041, China

**Keywords:** severe acute pancreatitis, isorhamnetin, mitochondrial dysfunction, HtrA2, KDM5B

## Abstract

Severe acute pancreatitis (SAP), a widespread inflammatory condition impacting the abdomen with a high mortality rate, poses challenges due to its unclear pathogenesis and the absence of effective treatment options. Isorhamnetin (ISO), a naturally occurring flavonoid, demonstrates robust antioxidant and anti-inflammatory properties intricately linked to the modulation of mitochondrial function. However, the specific protective impact of ISO on SAP remains to be fully elucidated. In this study, we demonstrated that ISO treatment significantly alleviated pancreatic damage and reduced serum lipase and amylase levels in the mouse model of SAP induced by sodium taurocholate (STC) or L-arginine. Utilizing an in vitro SAP cell model, we found that ISO co-administration markedly prevented STC-induced pancreatic acinar cell necrosis, primarily by inhibiting mitochondrial ROS generation, preserving ATP production, maintaining mitochondrial membrane potential, and preventing the oxidative damage and release of mitochondrial DNA. Mechanistically, our investigation identified that high-temperature requirement A2 (HtrA2) may play a central regulatory role in mediating the protective effect of ISO on mitochondrial dysfunction in STC-injured acinar cells. Furthermore, through an integrated approach involving bioinformatics analysis, molecular docking analysis, and experimental validation, we uncovered that ISO may directly impede the histone demethylation activity of KDM5B, leading to the restoration of pancreatic HtrA2 expression and thereby preserving mitochondrial function in pancreatic acinar cells following STC treatment. In conclusion, this study not only sheds new light on the intricate molecular complexities associated with mitochondrial dysfunction during the progression of SAP but also underscores the promising value of ISO as a natural therapeutic option for SAP.

## 1. Introduction

Acute pancreatitis (AP) is recognized as a prevalent acute inflammatory disorder within the abdominal cavity [1,2]. When AP advances to its more severe manifestation, known as severe acute pancreatitis (SAP), it has the potential to induce systemic inflammation and lead to multiple organ failure [3]. Notably, the pathogenesis of SAP, characterized by a high incidence and significant mortality [4], remains poorly understood. The current lack of specific and effective pharmaceutical interventions is highly unsatisfactory, as existing modalities for treating pancreatitis primarily offer supportive care [5]. Therefore, it is crucial to comprehensively understand the pathogenic mechanisms of SAP and develop effective treatment strategies.

Mitochondria, as the primary energy producers, play a crucial role in the physiology and pathology of the exocrine pancreas [6]. Following pancreatic injury, the excessive influx of Ca^2+^ and increased generation of reactive oxygen species (ROS) within the pancreatic acinar cells leads to mitochondrial depolarization, ATP depletion and oxidative damage to mitochondrial DNA (mtDNA) [7] These mitochondrial dysfunctions subsequently activate a series of cascades associated with programmed cell death and inflammatory signaling, ultimately leading to pancreatic necrosis and systemic inflammation [8]. Targeted restoration of mitochondrial functional homeostasis has become an emerging strategy for drug development in SAP [9].

Mitochondrial quality control proteins, including Parkin, PTEN-induced kinase 1 (PINK1), and high-temperature requirement A2 (HtrA2), have emerged as pivotal regulators of mitochondrial function in the context of SAP [10,11]. During SAP, the expression of Parkin and PINK1 in the pancreas increases, playing an autonomous protective role by promoting mitochondrial autophagy in pancreatic cells [11]. In contrast, the expression of HtrA2 is inhibited in damaged acinar cells. Pharmacologically enhancing HtrA2 expression in these compromised pancreatic acinar cells effectively restores mitochondrial function, thereby mitigating acinar cell necrosis and systemic inflammation [10]. This suggests that HtrA2 holds potential as a therapeutic target for regulating mitochondrial function in the treatment of SAP. Nevertheless, the specific regulatory mechanism inhibiting HtrA2 expression in damaged pancreatic tissue remains elusive.

Quercetin is a natural flavonoid extracted from various plants, exhibiting significant anti-pancreatitis activity by inhibiting intracellular ROS generation and inflammatory signals in damaged pancreatic acinar cells [12,13]. Isorhamnetin (ISO), a methylated derivative of quercetin, also demonstrates outstanding antioxidant and anti-inflammatory properties, with extensive studies highlighting its therapeutic potential in cardiovascular and inflammatory diseases [14]. However, whether ISO holds potential for treating pancreatitis has not been reported. Recent studies suggest that the antioxidative and anti-inflammatory effects of ISO are primarily linked to its direct regulation of mitochondrial functional homeostasis [15,16], such as mitochondrial biogenesis, ATP production, ROS generation, mitochondrial membrane potential, and the opening of the mitochondrial permeability transition pore (mPTP). Given the critical role of mitochondrial functional homeostasis in pancreatitis progression, we hypothesize that ISO may alleviate mitochondrial dysfunction in impaired pancreatic acinar cells, providing therapeutic potential in SAP.

In this study, we evaluated changes in pancreatic histology, serum amylase and lipase activities and pancreatic acinar cell necrosis to elucidate the impact of ISO on SAP-induced damage at both cellular and systemic levels. Assessment of changes in mitochondrial membrane potential, ATP production, mitochondiral ROS generation, oxidative damage and the release of mtDNA revealed that the protective effect of ISO against SAP is likely attributed to the preservation of mitochondrial function in damaged acinar cells. Mechanistically, through integrating bioinformatics analysis and experimental validation, we emphasized that ISO may restore mitochondrial dysfunction by directly inhibiting lysine-specific demethylase 5B (KDM5B) activity, thereby reinstating KDM5B-mediated transcriptional repression of the *HtrA2* gene.

## 2. Results

### 2.1. ISO Treatment Mitigates STC and L-Arginine-Induced Pancreatic Injury

**Figure 1:**
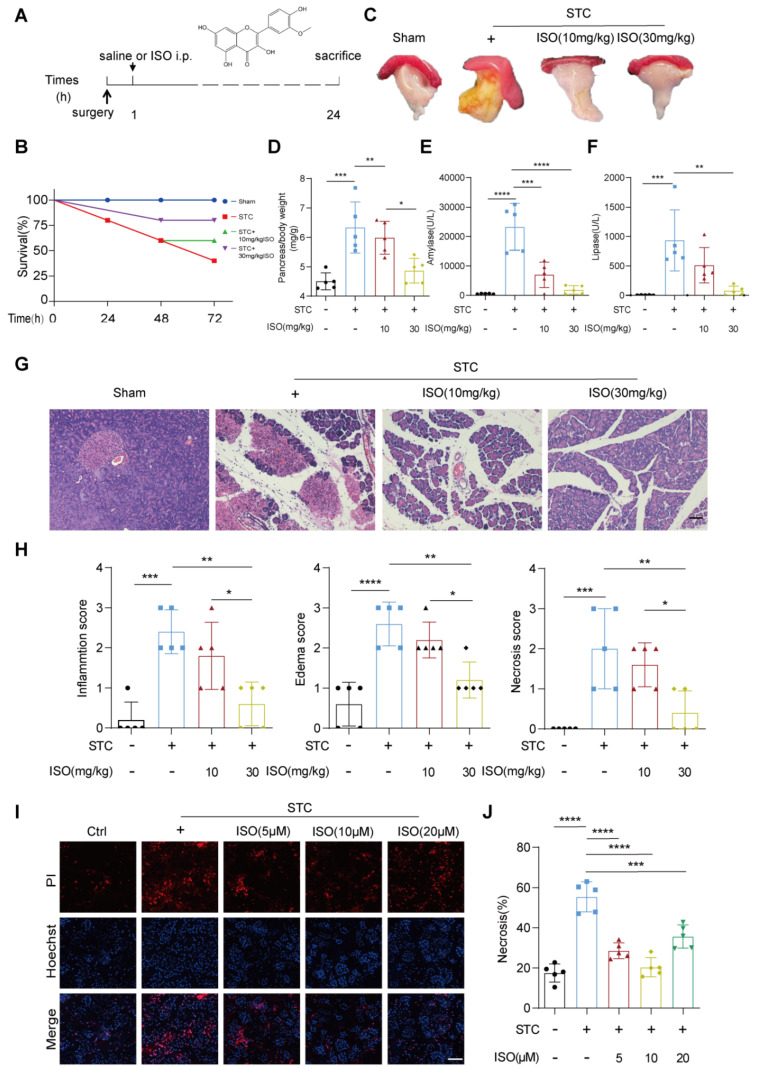
Protective effects of ISO against STC-induced SAP. (**A**) Intraperitoneal administration of saline or ISO (10, 30 mg/kg) one hour after SAP induction. (**B**) In the STC-induced SAP mouse model, the survival rate of SAP mice was calculated at different times (0, 24, 48, 72 h). (**C**) Representative images of freshly harvested pancreatic tissues. (**D**) Ratio of pancreatic weight to body weight at the time of sampling. *n* = 5. (**E**,**F**) Levels of serum amylase and lipase 24 h after SAP induction. *n* = 5. (**G**) Representative H&E images of pancreatic tissue sections, scale bars = 50 μm. (**H**) Histological scoring of inflammatory infiltration, edema and necrosis in pancreatic tissues to evaluate the extent of pancreatic injury. *n* = 5. (**I**) Representative images of primary pancreatic acinar cells stained with PI (red) and Hoechst 33342 (blue), scale bars = 50 μm. (**J**) Quantification of the percentage of PI-positive primary pancreatic acinar cells using Image J, *n* = 5. All data are presented as mean ± SD. Statistical significance was assessed using one-way ANOVA followed by Tukey’s multiple comparison test. * *p* < 0.05, ** *p* < 0.01, *** *p* < 0.001, **** *p* < 0.0001.

To examine the protective effect of ISO on SAP, we initially utilized a mouse model of SAP induced by a retrograde injection of a 3.5% solution of STC into the pancreatic duct, followed by intraperitoneal injection of either saline or varying doses of ISO (10 mg/kg and 30 mg/kg) one hour later (Figure 1A). Analysis of survival rates revealed a significant reduction in STC-induced mortality among mice receiving high-dose ISO treatment compared to those treated with saline alone (80% vs. 40%) (Figure 1B). Furthermore, we assessed pancreatic damage through the gross observation of the pancreas, determination of pancreas-to-body weight ratio, measurement of serum lipase and amylase activities and histopathological examination. ISO treatment notably decreased STC-induced pancreatic edema and yellow pus-like secretions on the pancreatic surface, as well as the increase in pancreas-to-body weight ratio in STC-treated mice (Figure 1C,D). Additionally, ISO administration effectively attenuated the elevation of serum amylase and lipase levels induced by STC injection (Figure 1E,F). Remarkably, significant improvement in histopathological changes in an STC-injected pancreas, including inflammatory cell infiltration, pancreatic edema and necrosis, was observed only with high-dose ISO treatment (30 mg/kg) (Figure 1G,H). Moreover, histological examination of lung tissue revealed a substantial amelioration in pancreatitis-associated acute lung injury in the high-dose ISO treatment group (Figure S1). To further investigate whether ISO prevents SAP by directly inhibiting STC-induced pancreatic acinar cell death, we treated primary pancreatic acinar cells with 5 mM STC to induce an in vitro model of SAP. Subsequent examination demonstrated that ISO administration significantly mitigated STC-induced acinar cell necrosis, with the most notable inhibitory effect observed at a concentration of 10 μM (Figure 1I,J).

In addition, to broaden our understanding of ISO’s therapeutic potential beyond biliary SAP, we conducted additional experiments using the L-arginine-induced experimental model of SAP. Both low (10 mg/kg) and high (30 mg/kg) doses of ISO treatment effectively alleviated the elevation of serum amylase and lipase levels induced by L-arginine, alongside improvements in pancreatic histopathological changes (Figure S2A–F). Similarly, ISO treatment mitigated L-arginine-induced acinar cell necrosis in an in vitro SAP model (Figure S2G,H). Collectively, these comprehensive findings underscore the universal protective potential of ISO against SAP.

### 2.2. ISO Administration Improves ROS Generation and Mitochondrial Dysfunction in STC-Treated Primary Pancreatic Acinar Cells

**Figure 2:**
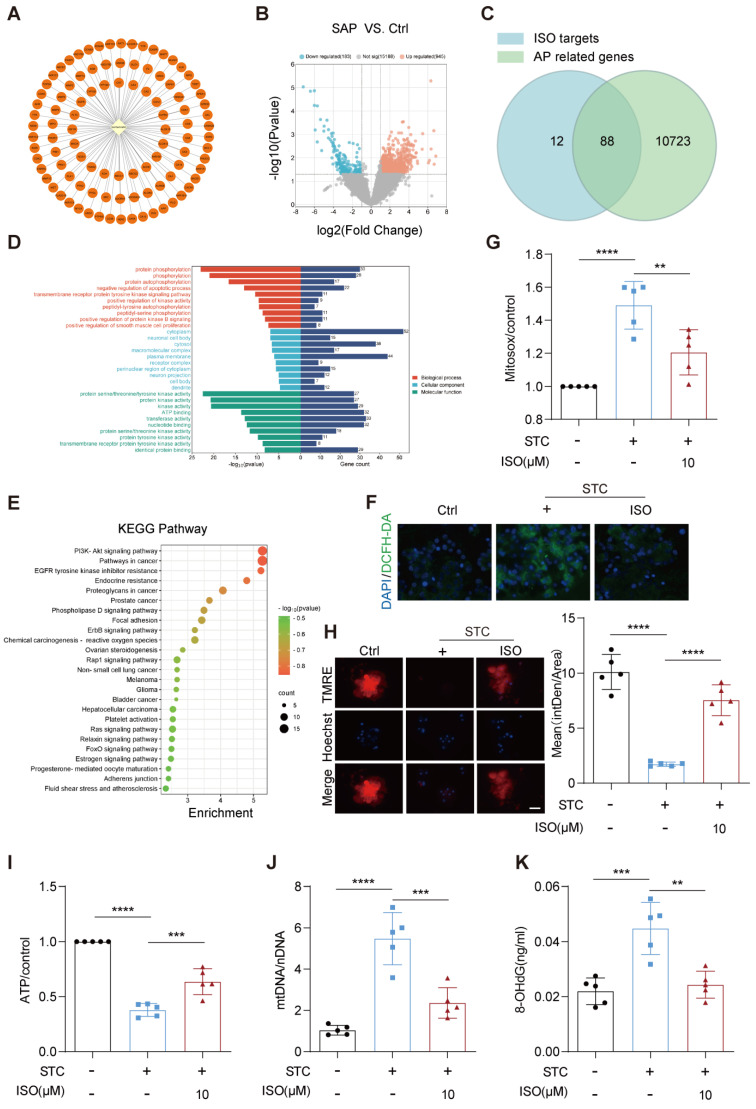
ISO-inhibited STC-induced oxidative stress and mitochondrial dysfunction. (**A**) Pharmacological target diagram of ISO. (**B**) Volcano plot of DEGs identified from the NCBI GEO database. (**C**) Venn diagram illustrating the targets of ISO in SAP. (**D**) GO enrichment analysis. (**E**) Bubble plot depicting the top 25 pathways based on KEGG enrichment analysis. (**F**) DCFH-DA fluorescence staining for measuring intracellular ROS levels in primary pancreatic acinar cells, scale bars = 20 μm, *n* = 5. (**G**) MitoSOX fluorescence staining for assessing mitochondrial ROS levels in primary pancreatic acinar cells, *n* = 5. (**H**) TMRE fluorescence staining to assess ΔΨm in primary pancreatic acinar cells, scale bars = 20 μm, *n* = 5. (**I**) ATP assay kit used to measure ATP levels in primary pancreatic acinar cells. *n* = 5. (**J**) qPCR analysis to determine the ratio of mtDNA to nDNA in the cytoplasm. *n* = 5. (**K**) ELISA measurement of Ox-mtDNA concentration in primary pancreatic acinar cells. *n* = 5. All data are presented as mean ± SD. Statistical significance was assessed using one-way ANOVA followed by Tukey’s multiple comparison test. ** *p* < 0.01, *** *p* < 0.001, **** *p* < 0.0001.

To unravel the intrinsic mechanisms behind the therapeutic effect of ISO on SAP, we conducted a comprehensive bioinformatics analysis encompassing potential pharmacological targets of ISO and genes linked to acute pancreatitis. A total of 100 potential pharmacological targets of ISO were identified based on the Swiss Target Prediction and TCMSP databases (Figure 2A). Acute pancreatitis-related genes were extracted from the Genecards database and the GEO (GSE161945) database, culminating in a total of 10,811 genes obtained (Figure 2B). Through the intersection of ISO targets and acute pancreatitis-related genes, we identified 88 potential targets of ISO that contribute to its anti-acute pancreatitis effect (Figure 2C). GO and KEGG enrichment analysis showed that these 88 potential ISO targets were predominantly enriched in biological processes or pathways, such as negative regulation of apoptosis process, protein kinase activity and chemical carcinogenesis-reactive oxygen species (ROS) (Figure 2D,E).

Considering that the substantial production of intracellular ROS following acute pancreatic injury is the primary catalyst for subsequent intracellular protein kinase activation and programmed cell death [17], we first assessed the alterations in intracellular and mitochondrial levels of ROS using DCHF-DA and MitoSOX assay in the SAP cell model following ISO treatment. As shown in Figure 2F, ISO co-administration effectively prevented STC-induced elevation in intracellular ROS generation in pancreatic acinar cells. Likewise, STC-induced increase in mitochondrial ROS production was markedly mitigated by ISO treatment (Figure 2G). Furthermore, we observed that STC-induced mitochondrial injury, including the decline in mitochondrial membrane potential (ΔΨm), reduction in ATP production, oxidative damage to mtDNA and release of mtDNA in pancreatic acinar cells, were all significantly hindered by ISO administration (Figure 2H–K). Collectively, these results indicate that the therapeutic effects of ISO against SAP may be attributed to its crucial role in preventing ROS generation and subsequent mitochondrial dysfunction in injured pancreatic acinar cells.

### 2.3. HtrA2 Is Essential for ISO’s Protective Effect on STC-Induced Mitochondrial Dysfunction

**Figure 3:**
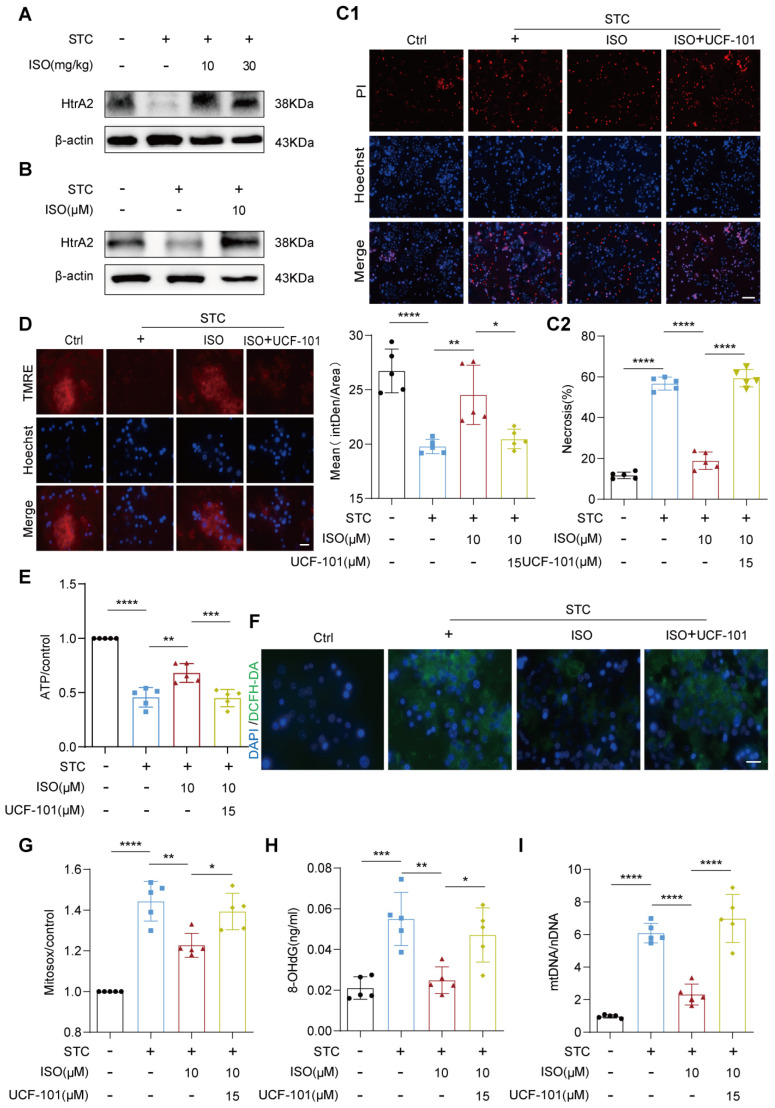
HtrA2 is essential for ISO’s protective effect on STC-induced mitochondrial dysfunction. (**A**,**B**) Western blot analysis of HtrA2 protein expression in pancreas and primary pancreatic acinar cells, *n* = 3. (**C1**,**C2**) Representative images of primary pancreatic acinar cells stained with PI (red) and Hoechst 33342 (blue), and quantification of the percentage of PI-positive primary pancreatic acinar cells using Image J, scale bars = 50 μm., *n* = 5. (**D**) TMRE fluorescence staining to assess ΔΨm in primary pancreatic acinar cells, scale bars = 20 μm, *n* = 5. (**E**) ATP assay kit used to measure ATP levels in primary pancreatic acinar cells, *n* = 5. (**F**) DCFH-DA fluorescence staining for measuring intracellular ROS levels in primary pancreatic acinar cells, scale bars = 20 μm, *n* = 5. (**G**) MitoSOX fluorescence staining for assessing mitochondrial ROS levels in primary pancreatic acinar cells, scale bars = 20 μm, *n* = 5. (**H**) ELISA measurement of Ox-mtDNA concentration in primary pancreatic acinar cells, *n* = 5. (**I**) qPCR analysis to determine the ratio of mtDNA to nDNA in the cytoplasm, *n* = 5. All data are presented as mean ± SD. Statistical significance was assessed using one-way ANOVA followed by Tukey’s multiple comparison test. * *p* < 0.05, ** *p* < 0.01, *** *p* < 0.001, **** *p* < 0.0001.

Previous studies have underscored the crucial protective role of ISO in regulating various processes related to mitochondrial quality control [15,16]. We next examined the effect of ISO treatment on the expression of HtrA2, a key mitochondrial quality control protein that is inhibited during SAP [10]. Western blotting analysis revealed a substantial increase in HtrA2 protein expression in the pancreas injected with STC following ISO administration (Figure 3A). Moreover, a notable restoration of HtrA2 protein expression was also observed in STC-treated primary acinar cells in vitro after ISO administration (Figure 3B).

To further explore the involvement of HtrA2 in mediating the protective effects of ISO on SAP, we employed the specific HtrA2 inhibitor, UCF-101, and assessed its impact on acinar cell necrosis, mitochondrial function, and ROS generation following ISO co-incubation. PI/Hoechst staining revealed that the addition of UCF-101 (15 μM) effectively abrogated the beneficial effect of ISO on STC-induced acinar cell necrosis (Figure 3C1,C2). Similarly, UCF-101 treatment significantly hindered the ISO-mediated recovery of ΔΨm (Figure 3D) and ATP production (Figure 3E) in STC-treated primary acinar cells, while also suppressing mitochondrial ROS generation (Figure 3F,G), alleviating mtDNA oxidative damage (Figure 3H) and preventing its release (Figure 3I). Collectively, our findings suggest that the protective effects of ISO against ROS generation and mitochondrial dysfunction in injured acinar cells likely involve direct modulation of the expression of the mitochondrial quality control protein HtrA2.

### 2.4. KDM5B Regulates HtrA2 Protein Expression in Pancreatic Acinar Cells during SAP

HSF1 and p53 are pivotal transcription factors recognized for their specific binding to the *HtrA2* promoter, thereby triggering the transcriptional activation of *HtrA2* [18,19]. Interestingly, our observations revealed a notable increase in the protein levels of HSF1 and p53 in STC-treated pancreatic acinar cells, accompanied by a decrease in the mRNA levels of *HtrA2* (Figure S3). This suggests that the reduced expression of HtrA2 in damaged acinar cells may be governed by regulatory mechanisms other than HSF1 and p53.

**Figure 4:**
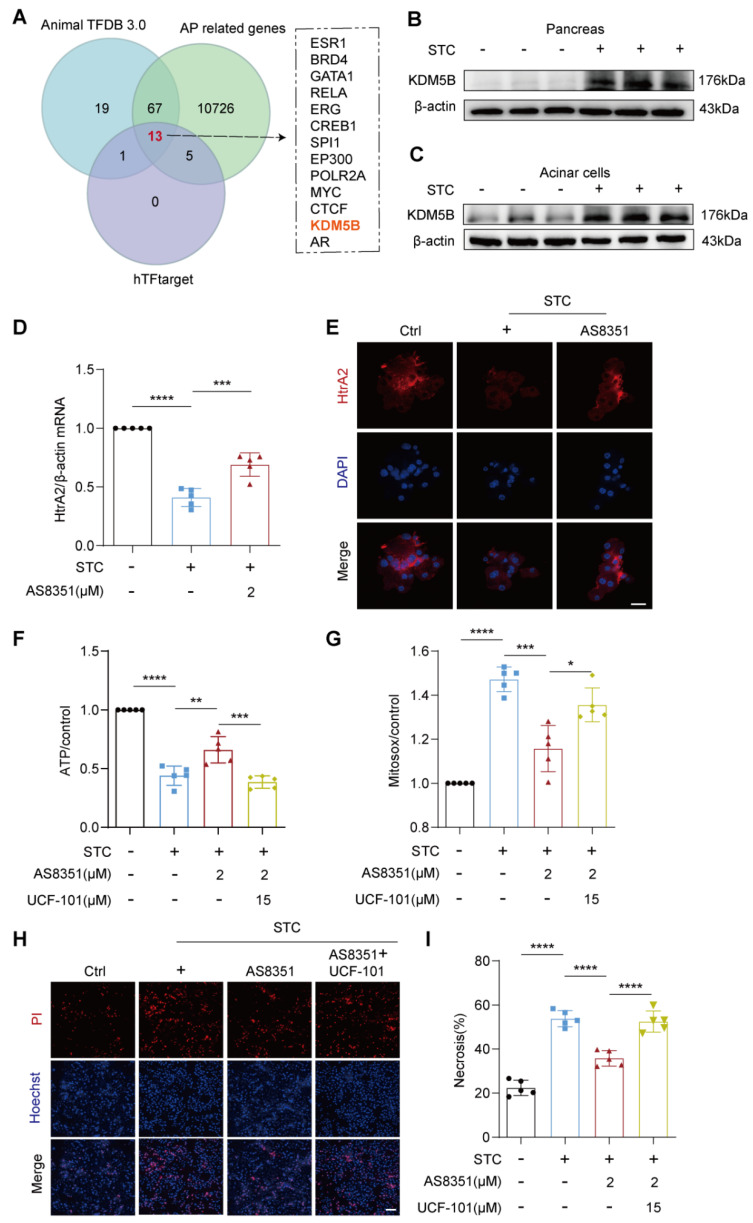
KDM5B suppressed expression of HtrA2. (**A**) Intersection between predicted transcription factors regulating HtrA2 and AP related genes. (**B**) Western blot analysis of KDM5B protein expression in pancreas, *n* = 3. (**C**) Western blot analysis of KDM5B protein expression in primary pancreatic acinar cells, *n* = 3. (**D**) The mRNA level of HtrA2 was detected by quantitative RT-PCR, *n* = 5. (**E**) Immunofluorescence staining for detecting HtrA2 protein expression in primary pancreatic acinar cells, scale bars = 100 μm, *n* = 5. (**F**) ATP assay kit used to measure ATP levels in primary pancreatic acinar cells, *n* = 5. (**G**) MitoSOX fluorescence staining for assessing mitochondrial ROS levels in primary pancreatic acinar cells, scale bars = 20 μm, *n* = 5. (**H**) Representative images of primary pancreatic acinar cells stained with PI (red) and Hoechst 33342 (blue), scale bars = 50 μm. (**I**) Quantification of the percentage of PI-positive primary pancreatic acinar cells using Image J, *n* = 5. All data are presented as mean ± SD. Statistical significance was assessed using one-way ANOVA followed by Tukey’s multiple comparison test. * *p* < 0.05, ** *p* < 0.01, *** *p* < 0.001, **** *p* < 0.0001.

By integrating the predicted transcription factors associated with *HtrA2* from the hTFtarget and AnimalTFDB databases, along with the aforementioned AP-related genes (GSE161945), we identified thirteen potential transcription factors with likely regulatory roles in pancreatic HtrA2 expression during SAP (Figure 4A). Notably, among these candidates, KDM5B emerges as a significant epigenetic regulator known for its role in repressing the expression of multiple genes via H3K4 demethylation. Building upon this insight, we investigated the protein level changes of KDM5B in the pancreas of STC-induced SAP mice. Our findings unveiled a notable elevation in KDM5B protein levels in damaged pancreases (Figure 4B). Further examination using an in vitro SAP cell model confirmed that STC triggers an increase in KDM5B levels within damaged acinar cells (Figure 4C), underscoring the potential role of heightened KDM5B as a primary driver of reduced HtrA2 expression during SAP.

To further elucidate the regulatory role of KDM5B on HtrA2 expression and HtrA2-mediated mitochondrial function, we intervened in STC-treated pancreatic acinar cells using the KDM5B-specific inhibitor AS8351 and examined its impact on HtrA2 expression, mitochondrial ATP and ROS production, as well as acinar cell necrosis. qPCR and immunofluorescence data revealed that AS8351 administration significantly increased both the mRNA and protein levels of HtrA2 in STC-treated acinar cells (Figure 4D,E). However, the AS8351-induced restoration of mitochondrial ATP production (Figure 4F), reduction in ROS generation (Figure 4G) and mitigation of acinar cell necrosis (Figure 4H,I) were all nullified when co-administered with the HtrA2 inhibitor, UCF-101. Therefore, our findings underscore the potentially critical regulatory role of KDM5B in pancreatic HtrA2 expression and its associated mitochondrial quality control processes during SAP.

### 2.5. ISO Functions as a Potential Inhibitor for KDM5B

**Figure 5:**
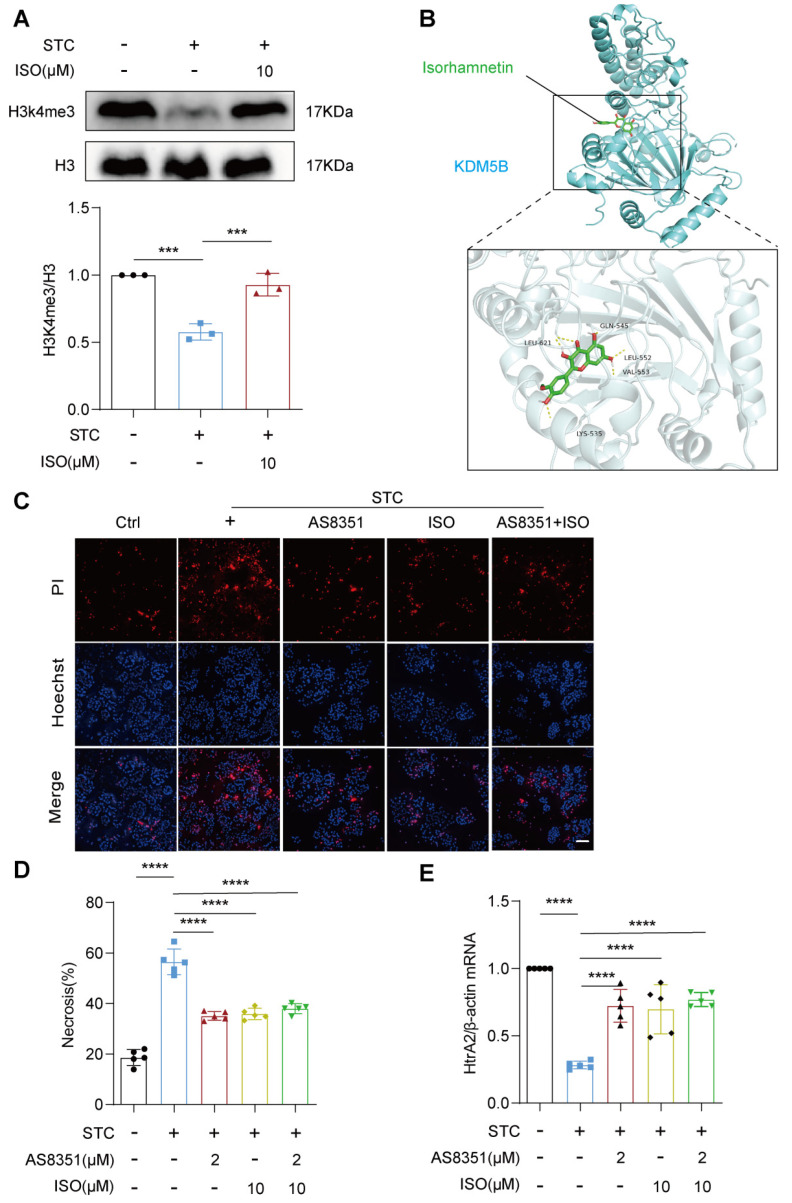
ISO functions as a potential inhibitor for KDM5B. (**A**) Western blot analysis of ISO’s impact on H3K4me3 protein expression, *n* = 3. (**B**) Representative visualized molecular binding model of ISO and KDM5B. (**C**,**D**) Representative images of primary pancreatic acinar cells stained with PI (red) and Hoechst 33342 (blue), and quantification of the percentage of PI-positive primary pancreatic acinar cells using Image J, scale bars = 50 μm, *n* = 5. (**E**) The mRNA level of HtrA2 was detected by quantitative RT-PCR, *n* = 5. All data are presented as mean ± SD. Statistical significance was assessed using one-way ANOVA followed by Tukey’s multiple comparison test. *** *p* < 0.001, **** *p* < 0.0001.

To explore the mechanism by which ISO facilitates the restoration of HtrA2 protein expression, we investigated whether this effect is mediated through the direct modulation of histone demethylation activity by KDM5B. Initially, we examined the impact of ISO treatment on genome-wide H3K4me3 signals in acinar cells treated with STC. Western blotting data showed that ISO administration significantly recovered STC-induced reduction in H3K4me3 protein levels (Figure 5A). Molecular docking analysis indicated a robust binding capacity (−5.98 kcal/mol) between ISO and KDM5B (Figure 5B), suggesting that ISO may function as a natural inhibitor for KDM5B. PI/Hoechst data demonstrated that additional treatment with ISO did not confer further protection against acinar cell necrosis in the context of KDM5B inhibition in STC-treated acinar cells (Figure 5C,D). Moreover, treatment with AS8351 in STC-induced SAP mice and acinar cells effectively mimicked the protective effects of ISO on pancreatic injury, as evidenced by improvements in pancreatic pathological indicators, serum amylase and lipase levels and STC-induced acinar cell necrosis (Figure S4). Additionally, examination of changes in HtrA2 mRNA levels revealed that ISO treatment did not exert an additional effect on AS8351-mediated restoration of *HtrA2* gene expression in response to STC treatment (Figure 5E). These collective findings suggest that ISO’s protective effect on SAP may stem from its direct inhibition of KDM5B’s histone demethylation activity, thereby reactivating HtrA2 gene transcription and subsequently restoring mitochondrial function.

## 3. Discussion

Monomeric components extracted from herbal medicines, particularly flavonoids, have exhibited considerable potential in treating inflammatory diseases owing to their robust antioxidant and anti-inflammatory attributes [20,21,22]. In the present study, we demonstrated for the first time that ISO, a natural flavonoid, exerts a significant protective effect on SAP, mainly by ameliorating STC-induced pancreatic mitochondrial dysfunction and subsequent acinar cell necrosis. At the molecular level, our investigation uncovered that ISO directly hinders the histone demethylation activity of KDM5B. This inhibition, in turn, leads to the restoration of pancreatic HtrA2 expression, thereby preserving mitochondrial function in STC-treated acinar cells.

The protective effects of quercetin in experimental models of pancreatitis have been extensively demonstrated over the past decade [12,23,24]. Quercetin intervention notably restrained cerulein-induced increases in serum amylase and lipase levels, while concurrently diminishing the expression and secretion of pro-inflammatory factors such as TNF-α and IL-6 in the injured pancreas [13]. As the methylated metabolite of quercetin, the therapeutic potential of ISO in SAP has not been explored. Our study revealed that the administration of relatively high doses (30 mg/kg) of ISO effectively mitigated STC- or L-arginine-induced pancreatic damage, manifesting in improved pancreatic structure and function and a reduction in acinar cell necrosis. This prompts the intriguing question of whether the previously observed protective effects of quercetin in pancreatitis are predominantly mediated by its metabolite, ISO. Unfortunately, unlike quercetin, which has undergone extensive clinical trials confirming its safety and potential efficacy in treating inflammatory diseases [25,26,27], ISO has not yet been tested in clinical trials in human. Nevertheless, considering ISO’s status as the primary metabolite of quercetin, concerns about its safety may be minimal. However, further experiments and clinical studies are urgently needed to determine whether ISO is the main contributor to the anti-inflammatory effects of quercetin. Such insights could pave the way for more effective treatment options for severe inflammatory diseases, including SAP.

Mitochondrial injury stands at the core of SAP pathogenesis [28]. The heightened production of mitochondrial ROS in compromised acinar cells initiates oxidative damage to mtDNA and triggers the opening of the mPTP [29]. Consequently, this cascade leads to the intracellular release of mtDNA, activating programmed necrotic processes [30]. Moreover, diminished ATP production resulting from mitochondrial damage exacerbates the impairment of pancreatic acinar cell secretory function, fostering acinar cell necrosis and escalating disease severity [31]. Our investigation, integrating bioinformatics analysis and experimental validation, delineates that the alleviative impact of ISO on STC-induced acinar cell necrosis primarily stems from the inhibition of mitochondrial ROS generation and the preservation of ATP production and ΔΨm, indicating a potential regulatory role of ISO on mitochondrial injury during SAP. Echoing our findings, earlier studies in cardiovascular and inflammatory diseases have highlighted ISO’s capacity to exert anti-inflammatory and antioxidant effects by directly modulating mitochondrial membrane potential, ROS generation, ATP production and mPTP opening [15,32]. However, the precise molecular mechanism through which ISO regulates mitochondrial function remains elusive. Previously, we demonstrated the pivotal protective role of the mitochondrial quality control protein HtrA2 in maintaining mitochondrial homeostasis during SAP. The pharmacological enhancement of HtrA2 expression has proven effective in restoring mitochondrial function and mitigating necrosis in STC-treated acinar cells [10]. In this study, we observed that the *HtrA2* gene was transcriptionally activated in both STC-injured pancreas and primary pancreatic acinar cells following ISO treatment. This implies that ISO’s regulatory effect on mitochondrial function may be linked to the activation of pancreatic HtrA2 expression. Nevertheless, the precise mechanism by which ISO orchestrates the transcriptional activation of *HtrA2* in a damaged pancreas remains an intriguing question.

HSF1 and p53 are recognized as key transcription factors for the *HtrA2* gene [18,19]. Intriguingly, our examination of protein expression in sodium taurocholate (STC)-treated acinar cells revealed a significant elevation in HSF1 and p53 levels within injured pancreatic acinar cells. These observations suggest that both STC-induced suppression and ISO-mediated restoration of pancreatic HtrA2 expression involve unidentified regulatory mechanisms.

By integrating transcription factor analysis with experimental validation, our study identified KDM5B as the sole epigenetic regulator among the 13 predicted transcription factors. KDM5B exhibited a substantial induction in damaged acinar cells with reduced genome-wide H3K4me3 signaling, a phenomenon effectively reversed by ISO administration. Furthermore, molecular docking analysis unveiled a binding potential between ISO and KDM5B, proposing that ISO-induced HtrA2 transcriptional activation may occur through direct binding to KDM5B. This interaction likely inhibits the histone demethylation activity of KDM5, consequently reactivating *HtrA2* gene transcription and facilitating the restoration of mitochondrial dysfunction. Supporting this notion, our experiments with the KDM5B-specific inhibitor AS8351 mirrored the protective effects of ISO on SAP. Strikingly, the beneficial impact of AS8351 was nullified upon HtrA2 inhibition, reinforcing the idea that ISO’s protective mechanism in SAP involves the modulation of KDM5B/HtrA2 signaling. Nonetheless, the molecular mechanism underlying the elevation of KDM5B protein levels in injured acinar cells remains unresolved and necessitates further exploration.

While our study sheds light on the potential regulatory role of ISO in the KDM5B/HtrA2 signaling pathway and its impact on SAP, several limitations warrant acknowledgment. The co-treatment results involving ISO and KDM5B inhibitor indicate that ISO may not elicit additional effects in enhancing *HtrA2* gene expression or protecting acinar cell necrosis. This suggests that the primary impact of ISO might be attributed to its unique regulatory role in the KDM5B/HtrA2 signaling pathway. To strengthen the credibility of our findings, it is crucial to conduct in vitro verification of the direct binding capacity between ISO and KDM5B. Techniques such as surface plasmon resonance (SPR) can offer more reliable molecular evidence, providing a deeper understanding of the molecular mechanisms that underlie ISO’s protective effects on SAP. Another noteworthy limitation lies in the absence of verification regarding the binding ability of KDM5B to the HtrA2 promoter region. Furthermore, the study did not investigate changes in the epigenetic modification levels of the *HtrA2* gene in damaged acinar cells, specifically the levels of H3K4me3 in its promoter region. To address these gaps in knowledge, employing techniques such as chromatin immunoprecipitation (ChIP) followed by high-throughput sequencing or PCR becomes essential. In the future, we aim to conduct further research on the aforementioned molecular mechanisms to provide deeper insights into the regulatory role of the KDM5B/HtrA2 signaling pathway in the pathogenesis of SAP.

## 4. Methods and Materials

### 4.1. Experimental Animals

Ethical approval for all aspects of the study was obtained from the Ethics Committee of West China Hospital, Sichuan University (Approval No. 20220221068). Male C57BL/6 mice, weighing approximately 25–30 g and aged 6–8 weeks, were sourced from SPF Biotechnology Co., Ltd. (Chengdu, China). The mice were acclimatized for one week under specific pathogen-free conditions, maintaining a temperature of 20–22 °C, relative humidity at 55% and a 12-h light–dark cycle.

### 4.2. SAP Model Establishment and Experimental Design

Prior to surgery, animals underwent a 12-h fasting period. Mice were randomly divided into four groups, each containing 5–8 animals.

STC-induced SAP mouse model: After anesthesia with 1% pentobarbital sodium, SAP was induced through retrograde injection into the pancreatic duct using 3.5% sodium taurocholate (STC). Isoproterenol (ISO) was administered intraperitoneally at doses of 10 mg/kg or 30 mg/kg, with saline serving as the control, given one hour after model induction. ISO was dissolved in DMSO and further diluted with saline. Cervical dislocation was performed to euthanize the mice 24 h post-administration, and serum, pancreatic and lung tissues were harvested.

L-arginine-induced SAP mouse model: Mice were subjected to intraperitoneal injections of 10% L-arginine to induce acute pancreatitis (4 g/kg body weight, pH = 7.0, with three injections at one-hour intervals). Subsequently, various doses of ISO (10 mg/kg or 30 mg/kg) were administered at the fourth hour, once daily for three consecutive days, and mice were sacrificed at 72 h.

### 4.3. Primary Pancreatic Acinar Cells Isolation

Primary pancreatic acinar cells were isolated following the procedure described by Yuan et al. [33]. In brief, after euthanasia and harvesting fresh pancreatic tissue, samples underwent digestion with Type IV collagenase at 37 °C for 19 min. Mechanical disruption separated the cells, which were then passed through a 100-micrometer cell strainer. Cell precipitation was achieved by centrifugation at 700 rpm for two minutes, and the cell pellet was resuspended in HEPES buffer.

### 4.4. Histological Examination

Pancreatic and lung tissues were fixed in 10% formalin, dehydrated and embedded in paraffin. Tissue sections were cut and stained with hematoxylin and eosin (H&E) for histological examination.

### 4.5. Serum Amylase and Lipase Measurement

Blood collected post-anesthesia via cardiac puncture into serum separator tubes was centrifuged at 3000 rpm for 10 min to obtain serum. A 50 μL aliquot of serum was diluted with distilled water to a final volume of 300 μL, and serum amylase and lipase levels were measured using a fully automated biochemical analyzer (Roche, Mannheim, Germany) following the manufacturer’s instructions.

### 4.6. Propidium Iodide (PI) Staining

Freshly isolated primary pancreatic acinar cells were treated with STC (final concentration 5  mM) and co-incubated with ISO, UCF-101 and AS8351 at 37 °C for 50 min. Cells were subsequently stained with Hoechst 33342 (50 μg/mL) and propidium iodide (PI, 1 μmol/mL). Images were captured using an upright fluorescence microscope (Axio Imager Z2, Zeiss, Oberkochen, Germany), and PI-positive cells were counted.

### 4.7. Potential ISO Targets Prediction

The SMILES information and PubChem CID of ISO were retrieved from the PubChem database (https://pubchem.ncbi.nlm.nih.gov/) and input into the SwissTargetPrediction database (http://www.swisstargetprediction.ch/) for target prediction. The data obtained from SwissTargetPrediction were designated as the ISO targets. In the NCBI GEO database (GSE161945) and Genecards database (https://www.genecards.org/), the keywords “severe acute pancreatitis” and “acute pancreatitis” were employed to retrieve genes associated with acute pancreatitis. After eliminating duplicates and false positives, the identified genes were cross-referenced with the ISO targets to identify potential therapeutic targets for ISO treatment in SAP. A network depicting “ISO targets” was constructed using Cytoscape software (version 3.9.1). Data from the above databases were obtained before 9 November 2023.

### 4.8. Targeted Pathway Analysis

To elucidate the signaling pathways affected by ISO in SAP, the 88 potential targets identified for ISO treatment in AP were imported into the DAVID Bioinformatics Resources for Gene Ontology (GO) and Kyoto Encyclopedia of Genes and Genomes (KEGG) enrichment analysis. Key pathways were visualized using bar and bubble plots on the web-based bioinformatics analysis platform (https://www.bioinformatics.com.cn/, accessed on 9 December 2023).

### 4.9. Intracellular ROS Examination

Reactive oxygen species (ROS) levels in primary pancreatic acinar cells were determined using the DCFH-DA probe (Beyotime, Shanghai, China). Freshly isolated cells were incubated with or without STC (final concentration 5 mM) along with ISO at 37 °C for 30 min. Subsequently, DCFH-DA was added at a final concentration of 10 μmol/L. After an additional 20 min of incubation in the dark at 37 °C, cells were washed twice with the HEPES buffer, and fluorescence signals were observed using a fluorescence microscope.

### 4.10. Mitochondrial Superoxide Assessment

MitoSOX Red staining was performed to detect the production of mitochondrial superoxide. Cells were collected, loaded with MitoSOX (MitoSOX Red, Yeasen, Shanghai, China) mitochondrial superoxide indicator for 30 min, and fluorescence intensity was measured by a fluorescence plate reader (Infinite 200 PRO, TECAN, Mannedorf, Switzerland).

### 4.11. Cellular Fractionation and Cytosolic mtDNA Measurement

Primary pancreatic acinar cells were lysed with mitochondria extraction buffer and centrifuged at 1000× *g* for 15 min at 4 °C [34]. The supernatant volume was calibrated based on protein concentration. Subsequently, the lysate was re-centrifuged (14,000 rpm, 4 °C, 30 min) to isolate the cytoplasmic fraction containing mtDNA and nDNA. DNA purification was performed using a MolPure Cell/Tissue DNA Kit (18700ES50, Yeasen, Shanghai, China) as per the manufacturer’s protocol. Quantitative PCR (q-PCR) was conducted to quantify mtDNA using primers for mitochondrial cytochrome c oxidase 1 (*COX1*), while nuclear DNA was measured using primers for *18S* ribosomal RNA. The ratio of mtDNA to nDNA copy number was calculated to assess the release of mtDNA into the cytoplasm. Primer sequences for mouse *COX1* and *18S* were as follows [35] (Sangon Biotech, Shanghai, China):

*18S*-forward: 5′-TAGAGGGACAAGTGGCGTTC-3′;

*18S*-reverse: 5′-CGCTGAGCCAGTCAGTGT-3′;

*Cox1*-forward: 5′-GCCCCAGATATAGCATTCCC-3′;

*Cox1*-reverse: 5′-GTTCATCCTGTTCCTGCTCC-3′;

### 4.12. RT-qPCR Analysis

The total RNA of was isolated using TRIzol™ reagent (Invitrogen, Carlsbad, CA, USA) according to the manufacturer’s instructions. Total RNA was reverse-transcribed using Hifair III 1st Strand cDNA Synthesis SuperMix (Yeasen, Shanghai, China). Real-time qPCR was performed using Hieff^®^ qPCR SYBR Green Master Mix Kit (Yeasen, Shanghai, China) on a Thermo Q6 Real-Time System, according to the manufacturer’s instructions. Gene expression relative to that of *β-actin* was analyzed for each sample using the 2^−ΔΔCt^ method. The primers were designed and synthesized as follows:

*HtrA2*-F: 5′-ATCTCCTTTGCCATCCCTTC-3′;

*HtrA2*-R: 5′-GGTCAGCATCATCACTCCAA-3′;

*β-actin*-F:5′-GTGACGTTGACATCCGTAAAGA-3′;

*β-actin*-R:5′-GCCGGACTCATCGTACTCC-3′;

### 4.13. 8-OHdG Production Measurement

Mitochondrial DNA was extracted from freshly isolated primary pancreatic acinar cells using a mitochondrial DNA extraction kit (Saint-Bio, Shanghai, China), following the manufacturer’s instructions. The concentration of mtDNA was determined by spectrophotometry; a lack of protein contamination was verified; and oxidation of mtDNA (Ox-mtDNA) was measured using an 8-OHdG assay kit (FineTest, Wuhan, China) to determine levels of 8-OHdG, a marker of oxidative DNA damage, relative to mtDNA concentration.

### 4.14. Western Blotting Analysis

Pancreatic tissue or isolated primary pancreatic acinar cells were thoroughly lysed using RIPA lysis buffer containing phosphatase and protease inhibitors, and equal amounts of protein were electromigrated and transferred onto a PVDF membrane. Primary antibodies for KDM5B (1:1000, Abcam, Cambridge, UK), H3K4me3 (1:1000, Abcam, Cambridge, UK), H3 (1:5000, Abcam, Cambridge, UK), HtrA2 (1:1000, Abclone, Wuhan, China), p53 (1:200, Santa Cruz Biotechnology, Santa Cruz, CA, USA) and HSF1 (1:1000, CAT: WL02759, Shenyang, China) were used, with image detection performed using an enhanced chemiluminescence (ECL) system (Bio-Rad, San Jose, CA, USA).

### 4.15. Immunofluorescence Staining

Isolated primary pancreatic acinar cells were incubated with STC for 50 min, fixed with 4% paraformaldehyde for 1 h, and incubated overnight at 4 °C with primary antibodies against HtrA2 (1:100, Abclone). The cells were then incubated in the dark with corresponding secondary antibodies (1 h, 37 °C). Finally, DAPI was used for nucleus counterstaining for 10 min, and images were captured with a fluorescence microscope (Nikon, Tokyo, Japan).

### 4.16. Molecular Docking

The 3D structure of the target protein was obtained from the Protein Data Bank (PDB, https://www.rcsb.org, accessed on 6 November 2023). Using PyMOL 2.5.2, water molecules and original ligands were removed, and the protein was prepared in AutoDock Tools 1.5.6 for molecular docking. Docking results were visualized using PyMOL 2.5.2.

### 4.17. Statistical Analysis

Data are presented as the mean ± standard deviation (SD). Statistical analysis were conducted using GraphPad Prism 8.0, employing two-sided Student’s unpaired *t*-tests or one-way analysis of variance (ANOVA) as appropriate. A significance level of *p* < 0.05 was considered statistically significant.

## 5. Conclusions

This study unveils, for the first time, the therapeutic promise of ISO in severe acute biliary pancreatitis, primarily attributed to its ability to ameliorate mitochondrial dysfunction and alleviate pancreatic acinar cell necrosis. Our findings highlight the pivotal role of KDM5B induction in damaged pancreatic acinar cells, leading to the repression of HtrA2 transcription and subsequent mitochondrial dysfunction. Additionally, our investigation reveals the potential of ISO to bind to KDM5B, suggesting its role as a natural inhibitor of KDM5B-mediated histone demethylation at the HtrA2 promoter. Collectively, these discoveries not only offer novel insights into the molecular intricacies associated with mitochondrial dysfunction during the progression of SAP but also open new avenues for the development of therapeutic strategies targeting mitochondrial dysfunction in severe acute biliary pancreatitis.

## Data Availability

This article and Supplementary Material contains all data from this study.

## Supplementary Materials

The following supporting information can be downloaded at: https://www.mdpi.com/article/10.3390/ijms25073784/s1.
