# Supplementary material for: Isorhamnetin Alleviates Mitochondrial Injury in Severe Acute Pancreatitis via Modulation of KDM5B/HtrA2 Signaling Pathway
Source: Int J Mol Sci. 2024 Mar 28;25(7):3784. doi: 10.3390/ijms25073784 (PMC11011973; doi:10.3390/ijms25073784)
Supplement: Supplementary file 1 [file ijms-25-03784-s001.zip › ijms-2899668-supplementary.pdf]

Supplementary Material

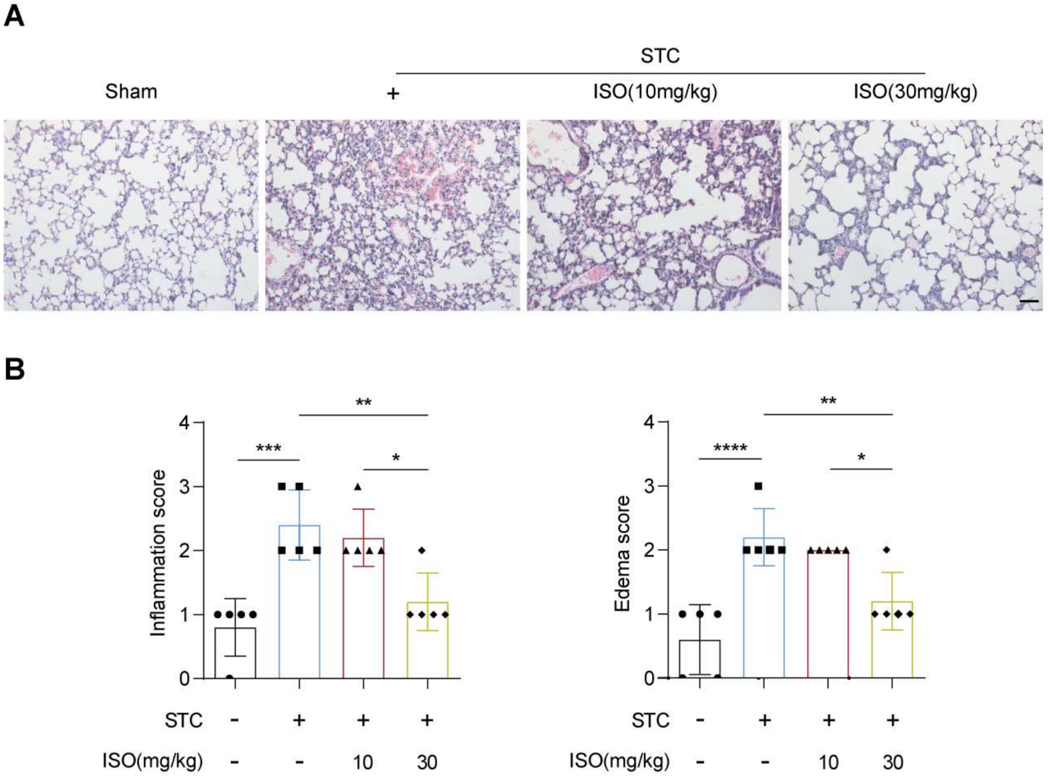

Figure. S1 ISO alleviates STC-ossociated lung injury. (A) Representative H&E images of lung tissue sections, scale bars = 50 μm. (B) Histological scoring of inflammation, edema in lung tissues to evaluate the extent of lung injury. n=5. All data are presented as mean ± SD. Statistical significance was assessed using one-way ANOVA followed by Tukey's multiple comparison test. $^{*}p < 0.05$, $^{**}p < 0.01$, $^{***}p < 0.001$, $^{****}p < 0.0001$.

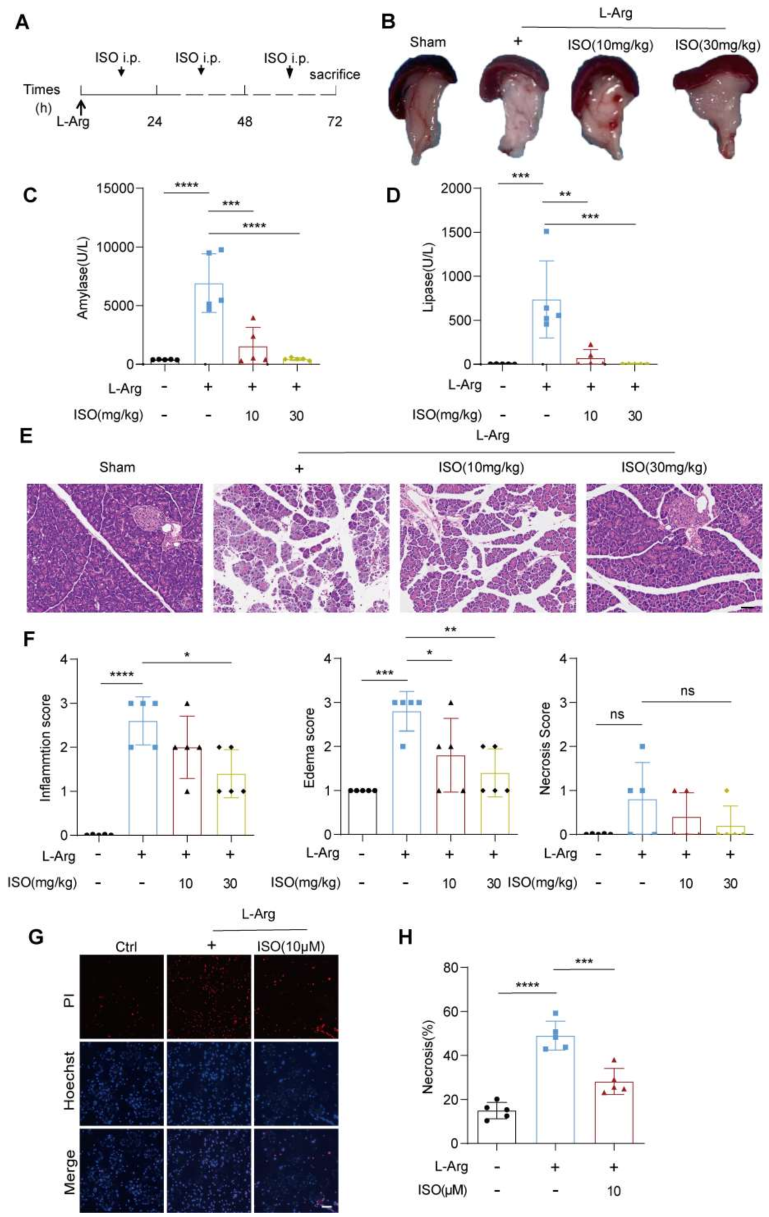

Fig. S2 Protective effects of ISO against L-Arg induced AP. (A) Intraperitoneal administration of saline or ISO (10, 30 mg/kg) four hours after AP induction. (B) Representative images of freshly harvested pancreatic tissues. (C, D) Levels of serum amylase and lipase 72 hours after AP induction. *n*=5. (E) Representative H&E images of pancreatic tissue sections, scale bars = 50 μm. (F) Histological scoring of inflammatory infiltration, edema, and necrosis in pancreatic tissues to evaluate the extent of pancreatic injury. *n*=5. (G) Representative images of primary pancreatic acinar cells stained with PI (red) and Hoechst 33342 (blue), scale bars = 50 μm. (H) Quantification of the percentage of PI-positive primary pancreatic acinar cells using Image J, *n*=5. All data are presented as mean ± SD. Statistical significance was assessed using one-way ANOVA followed by Tukey's multiple comparison test. ns = $p > 0.05$, $^{*}p < 0.05$, $^{**}p < 0.01$, $^{***}p < 0.001$, $^{****}p < 0.0001$.

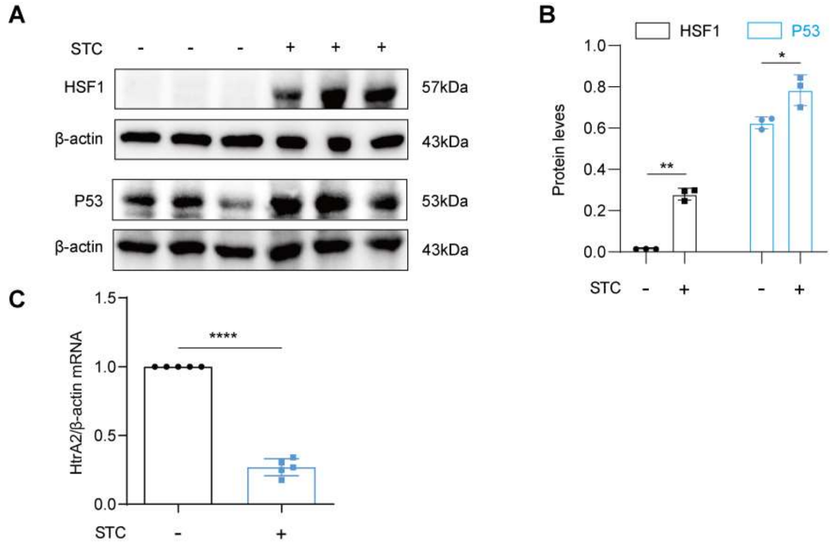

Figure. S3 HSF1 and P53 were upregulated in STC-induced SAP. (A, B) Western blot analysis of HSF1 and P53 protein expression in primary pancreatic acinar cells, n=3. (C) The mRNA level of HtrA2 was detected by quantitative RT-PCR, n=5. All data are presented as mean ± SD. Statistical significance was assessed using two-sided Student's unpaired t-tests. $^{*}p < 0.05$, $^{**}p < 0.01$, $^{****}p < 0.0001$.

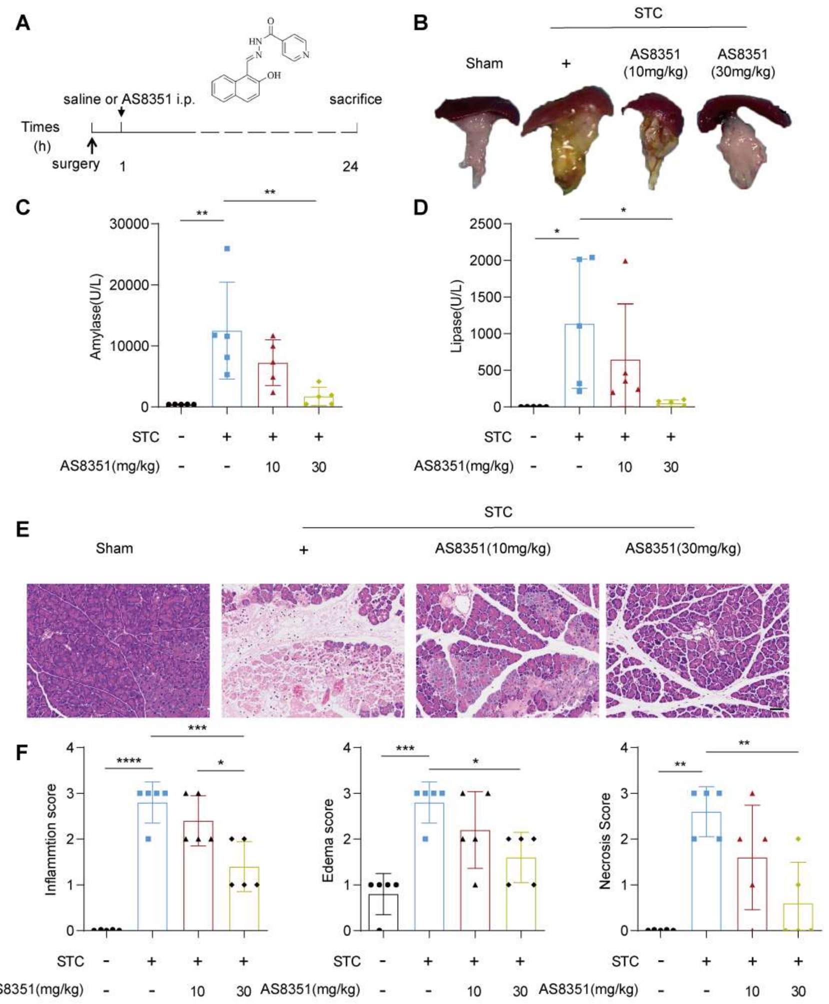

Fig. S4 Protective effects of AS8351 on STC-induced SAP. (A) Intraperitoneal administration of saline or AS8351 (10, 30 mg/kg) four hours after AP induction. (B) Representative images of freshly harvested pancreatic tissues. (C, D) Levels of serum amylase and lipase 24 hours after SAP induction. *n*=5. (E) Representative H&E images of pancreatic tissue sections, scale bars = 50 μm. (F) Histological scoring of inflammatory infiltration, edema, and necrosis in pancreatic tissues to evaluate the extent of pancreatic injury. *n*=5. All data are presented as mean ± SD. Statistical significance was assessed using one-way ANOVA followed by Tukey's multiple comparison test. $^{*}p < 0.05$, $^{**}p < 0.01$, $^{***}p < 0.001$, $^{****}p < 0.0001$.
